# Exploring the Impact of Extraplatelet Content on Fibrin-Based Scaffold Performance for Regenerative Therapies

**DOI:** 10.3390/ijms26135967

**Published:** 2025-06-21

**Authors:** Daniel Marijuán-Pinel, Jon Mercader-Ruiz, Maider Beitia, Pello Sánchez, Leonor López de Dicastillo, Sergio Gonzalez, João Espregueira-Mendes, Beatriz Aizpurua, Jaime Oraá, Diego Delgado, Mikel Sánchez

**Affiliations:** 1 Advanced Biological Therapy Unit, Hospital Vithas Vitoria, 01008 Vitoria-Gasteiz, Spain; daniel.marijuan@ucatrauma.com (D.M.-P.); jon.mercader@ucatrauma.com (J.M.-R.); maider.beitia@ucatrauma.com (M.B.); pello.sanchez@ucatrauma.com (P.S.); diego.delgado@ucatrauma.com (D.D.); 2 Arthroscopic Surgery Unit, Hospital Vithas Vitoria, 01008 Vitoria-Gasteiz, Spain; leonor.lopez@ucatrauma.com (L.L.d.D.); sergio.gonzalez@ucatrauma.com (S.G.); beatriz.aizpurua@ucatrauma.com (B.A.); jaime.oraa@ucatrauma.com (J.O.); 3 Clínica Espregueira—FIFA Medical Centre of Excellence, 4350-415 Porto, Portugal; jem@espregueira.com; 4 Dom Henrique Research Centre, 4350-415 Porto, Portugal; 5 School of Medicine, University of Minho, 4710-057 Braga, Portugal; 6 ICVS/3B’s-PT Government Associate Laboratory, 4710-057 Braga, Portugal; 7 3B’s Research Group—Biomaterials, Biodegradables and Biomimetics, University of Minho, Headquarters of the European Institute of Excellence on Tissue Engineering and Regenerative Medicine, Barco, 4805-694 Guimarães, Portugal

**Keywords:** platelet-rich plasma, fibrin scaffold, biomaterial, biomechanic, biodegradation

## Abstract

This study investigated the impact of increased extraplatelet content on the tissue regenerative capacity of platelet-rich plasma (PRP)-derived fibrin scaffolds. Comparative analyses were performed between a “balanced protein-concentrate plasma” (BPCP) and a standard PRP (sPRP), focusing on platelet and fibrinogen content, scaffold microstructure, and functional performance. Growth factor (GF) release kinetics from the scaffolds were quantified via ELISA over 10 days, while scaffold biomechanics were evaluated through rheological testing, indentation, energy dissipation, adhesion, and assessments of coagulation dynamics, biodegradation, swelling, and retraction. Microstructural analysis was conducted using scanning electron microscopy (SEM), with fiber diameter and porosity measurements. The results demonstrated that BPCP scaffolds released significantly higher amounts of GFs and total protein, especially beyond 24 h (* *p* < 0.05). Despite a delayed coagulation process (** *p* < 0.01), BPCP scaffolds exhibited superior structural integrity and cushioning behavior (* *p* < 0.05). SEM revealed thicker fibers in BPCP scaffolds (**** *p* < 0.0001), while adhesion and biodegradation remained unaffected. Notably, BPCP scaffolds showed reduced retraction after 24 h and maintained their shape stability over two weeks without significant swelling. These findings indicate that enhancing the extraplatelet content in PRP formulations can optimize fibrin scaffold performance. Further preclinical and clinical studies are warranted to evaluate the therapeutic efficacy of BPCP-derived scaffolds in regenerative medicine.

## 1. Introduction

Platelet-rich plasma (PRP) has gained considerable attention as a regenerative therapeutic tool in various medical fields such as wound healing, orthopedics, and sports medicine due to its capacity to promote tissue healing and repair [1,2,3]. PRP is a biological product derived from the patient’s own blood, enriched with platelets, growth factors (GFs) and bioactive proteins that play a crucial role in tissue healing and regeneration [1,4,5,6]. PRP formulations typically vary in terms of platelet concentration, fibrinogen content, leukocyte levels, and fibrin clot formation [7]. As such, understanding how different PRP formulations compare in terms of such properties is essential for optimizing their use in clinical settings.

The possibility of formulating PRP as a biomaterial scaffold offers a promising strategy to improve the therapeutic results of PRP. Today, different formulations of PRP as scaffold biomaterial are available. Using external activators such as CaCl_2_, thrombin or collagen, fibrin-based scaffolds are created by cleavage of the fibrinogen from the PRP itself [8,9]. Plasma-derived fibrin-based scaffolds have become the cornerstone of regenerative medicine due to their biocompatibility, biodegradability and ability to closely mimic the natural extracellular matrix. These scaffolds promote cell migration, proliferation and differentiation, processes vital for tissue regeneration. However, the efficacy of PRP-derived fibrin scaffolds depends on both the formulation of the PRP and the physical properties of the scaffold itself [10,11,12]. Studies show that the concentration of activators used, as well as the method of PRP procurement [13], influence the regenerative capacity of these biomaterials [14,15]. However, it has been shown that these PRP matrices lack a sustained GF release, in addition to exhibiting a low mechanical strength. Therefore, in order to improve the characteristics of PRP scaffolds, new formulations of PRP scaffolds are being developed, such as loading PRP into scaffolds made of synthetic or natural materials like chitosan or hyaluronic acid [9].

Among the properties of scaffolds, biomechanical performance is a key factor determining their success in regenerative medicine [16]. Indeed, in the context of orthopedic pathologies, these scaffolds are subjected to a variety of mechanical and contractile forces inherent to the tissue environment [17]. Thus, the stiffness, elasticity, and adhesion properties of fibrin scaffolds must be precisely tailored to match the specific mechanical demands of the target tissue. In addition, the physical behavior of fibrin scaffolds, such as their swelling and retraction, has significant implications for their performance in vivo. Swelling is a critical property that influences nutrient diffusion and cellular migration within the scaffold [18] and retraction capacity affects the scaffold’s ability to maintain its shape and volume during tissue integration [19,20,21]. Understanding these biomechanical and physical properties is essential for developing scaffolds that offer stable support throughout the regenerative process.

Moreover, fibrin-based scaffolds have the ability to encapsulate and release GFs in a controlled manner [22], prolonging the presence of these bioactive molecules at the injury site and, thus, facilitating sustained tissue regeneration. These GFs include platelet-derived growth factor (PDGF), vascular endothelial growth factor (VEGF), transforming growth factor-beta 1 (TGF-β1), and extraplatelet GFs such as hepatocyte growth factor (HGF) and insulin-like growth factor (IGF-1). They play crucial roles in cellular recruitment, proliferation, regulation of inflammation, angiogenesis, and collagen synthesis, all of which are essential for efficient tissue healing [4,5,23]. Indeed, the concentration of GFs and cytokines within the fibrin PRP clot is directly linked to its effectiveness [24,25].

Recent advancements in PRP formulations have led to the development of balanced-protein concentrate plasma (BPCP). Unlike standard PRP (sPRP) formulations, it not only has an increased platelet content but also an enhanced extraplatelet content such as platelet-external GFs IGF-1, HGF and FGF-2, and plasma-circulating proteins such as fibrinogen and alpha-2-macroglobulin (A2M) [26]. The incorporation of BPCP into fibrin scaffolds may therefore augment the regenerative capabilities of the scaffold, leading to improved outcomes in a variety of therapeutic applications, including tissue engineering and wound healing.

In this study, we examined two different PRP formulations, BPCP and sPRP, to evaluate their influence on fibrin scaffold properties. Overall, the goal of this study is to provide a comprehensive understanding of how the composition of PRP affects the performance of scaffolds for clinical use. To achieve that, this study aimed to quantify and compare the release profiles of key growth factors from sPRP and BPCP scaffolds. It also evaluated their biomechanical properties and, additionally, determined whether the higher extraplatelet content in BPCP enhances the structural and functional qualities of fibrin scaffolds. Our hypothesis is that the increase in extraplatelet molecules in BPCP leads to better biochemical and biomechanical characteristics when developing a fibrin scaffold for clinical use.

## 2. Results

### 2.1. BPCP Shows a Two-Fold Increase of Both Platelet and Fibrinogen Levels

Platelet and fibrinogen levels were measured in blood, sPRP and BPCP to compare their biochemical composition. Platelet analysis showed that the platelet content in sPRP (335.76 × 10^3^ platelets µL^−1^ ± 111.32) and BPCP (342.94 × 10^3^ platelets µL^−1^ ± 117.03) was twice that of blood basal levels (186.18 × 10^3^ platelets µL^−1^ ± 67.72), and these differences were statistically significant (**** *p* < 0.0001). Moreover, there was no significant difference in platelet concentration between sPRP and BPCP (*p* = 0.8763). Meanwhile, residual leukocytes and erythrocytes were below the detection limit. According to the UCS (Universal Coding System) for PRP studies described by Kon et al. [27], the products used in this study were 13-00-11 (Table 1).

As for fibrinogen content, no significant differences were found between blood (374.14 mg dL^−1^ ± 34.57) and sPRP (364.57 mg dL^−1^ ± 32.56) (*p* = 0.9718). In contrast, BPCP (718.71 mg dL^−1^ ± 55.95) showed twice the fibrinogen content compared to both blood (**** *p* < 0.0001) and sPRP (*p* = 0.0003) (Table 1).

### 2.2. BPCP Fibrin Scaffold Fibers Have a Wider Diameter and Smaller Pore Size than sPRP Scaffold Fibers

**Figure 1:**
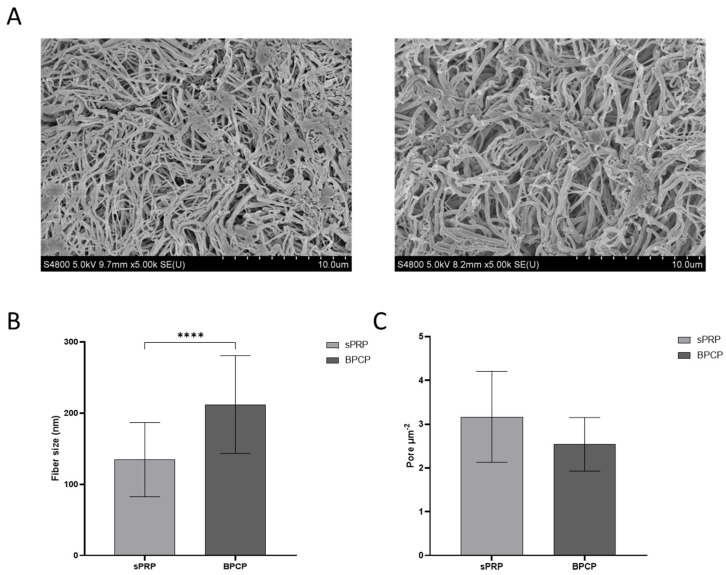
Morphological analysis of sPRP and BPCP scaffolds. (**A**) SEM images of sPRP (**left**) and BPCP (**right**). Scale bar 10 µm. (**B**) Fiber diameter size in sPRP and BPCP is expressed in nm. (**C**) Porosity is represented by the number of pores per µm^2^. Fiber diameter and number of pores were measured using the software ImageJ v 1.51W. Error bars = standard deviation (*n* = 4). Statistically significant differences were calculated using Student’s *t* test (**** *p* < 0.0001).

SEM images were used to analyze the inner structure of both types of scaffolds. Fibrin fibers from the two formulations, sPRP and BPCP, revealed distinct visual structural differences (Figure 1A). The sPRP scaffolds showed thinner and more dispersed fibrin fibers, with a higher number of visible pores throughout the structure. In contrast, the BPCP formulation displayed thicker fibrin fibers, resulting in a more robust and denser fibrin network.

These qualitative characteristics were quantified, and fiber diameter and porosity were measured. The results were consistent with those previously reported, since a significant difference was observed between the size of the fibers in the two formulations (**** *p* < 0.0001), being larger in the BPCP scaffold (Figure 1B). Moreover, a lower number of pores was observed in the BPCP samples (Figure 1C).

### 2.3. Biomechanical Behavior

#### 2.3.1. BPCP Shows a Deceleration of the Coagulation Process

**Figure 2:**
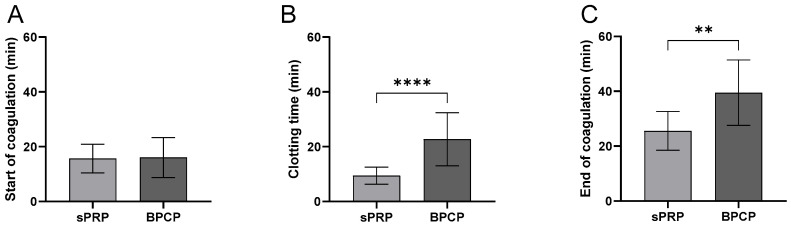
Coagulation kinetics of sPRP and BPCP scaffolds. The duration of the different phases of the clotting process is shown in the figure: start of coagulation (**A**), clotting process (**B**) and end of coagulation (**C**). Time is expressed in minutes in all graphs. Error bars = standard deviation (*n* = 9). Statistically significant differences were calculated using Student’s *t* test (** *p* < 0.01; **** *p* < 0.0001).

Regarding clotting time for scaffold formation, Figure 2 presents the coagulation parameters for the sPRP and BPCP formulations.

The start of coagulation (Figure 2A) did not show a significant difference between the sPRP and BPCP formulations in terms of the clotting starting process.

Clotting formation time (Figure 2B) was significantly longer in the BPCP than in the sPRP (**** *p* < 0.0001). The BPCP formulation exhibited a markedly higher mean clotting time, indicating a delayed coagulation process.

Finally, the end of coagulation (Figure 2C) was also significantly delayed in the BPCP group compared to the sPRP group (** *p* < 0.01). The mean value for BPCP was notably higher, suggesting a prolonged coagulation phase.

These findings suggest that the BPCP group exhibits delayed clotting kinetics compared to the sPRP group, particularly in terms of clotting time and the completion of coagulation.

#### 2.3.2. BPCP Exhibits a More Solid, Consistent and Stiffer Formulation

The rheological profile of PRP derived scaffolds was analyzed to determine their viscoelastic behavior under different deformation conditions. Key parameters such as ‘storage modulus’ (G′) and ‘loss modulus’ (G″) were calculated to obtain tanδ, which describes the viscoelasticity of the material (Table 2).

Both formulations showed a tanδ value below 1, meaning that both scaffolds have elastic (solid-like) behavior and the material will recover its original shape after deformation. Moreover, BPCP showed a lower liquidity behavior and thus greater elasticity and rigidity, as its tanδ value tended to decrease (Figure 2A).

**Figure 3:**
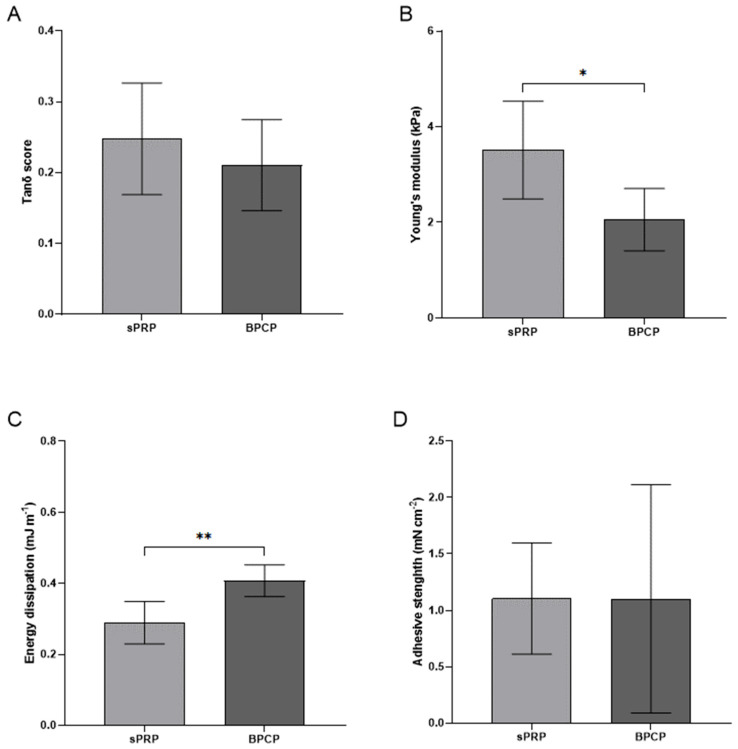
Mechanical properties of sPRP and BPCP scaffolds. The graphs show the sweep amplitude data showing the tanδ score (viscoelasticity) (**A**), Young’s modulus (stiffness) (**B**), the dissipation energy (cushioning) (**C**), the adhesion capacity (**D**) of the sPRP and BPCP formulations. Error bars = standard deviation (*n* = 4–7). Statistically significant differences were calculated using Student’s *t* test (* *p* < 0.05; ** *p* < 0.01).

The Young’s modulus data showed that the sPRP (3.51 kPa ± 1.02) had significantly greater stiffness than the BPCP formulation (2.06 kPa ± 0.65) (* *p* = 0.0147) (Figure 3B).

Furthermore, the BPCP composite dissipated energy of 0.4 mJ m^−1^ ± 0.045, presenting a significantly higher cushioning capacity than the sPRP formulation, which dissipated energy of 0.26 mJ m^−1^ ± 0.06 (** *p* = 0.0087) (Figure 3C).

Finally, no significant differences were observed between the two scaffolds in terms of the adhesion strength of BPCP (1.1 mN cm^−2^ ± 91.74) and sPRP (1.11 mN cm^−2^ ± 0.49) (*p* = 0.9966) (Figure 3D).

#### 2.3.3. Retraction and Swelling Capacity

**Figure 4:**
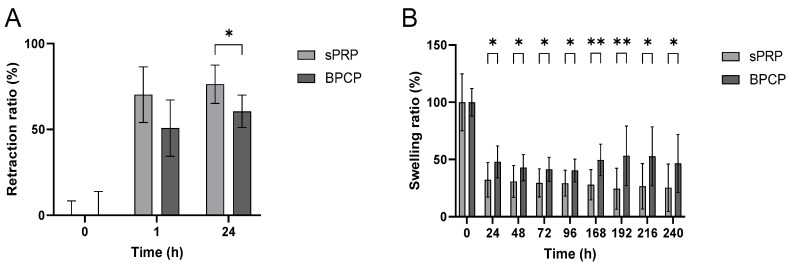
Retraction and swelling ratios of sPRP and BPCP scaffolds. The retraction ratio (%) of sPRP and BPCP scaffolds was measured up to 24 h (**A**), and swelling ratio (%) were determined up to 240 h (**B**). Error bars = standard deviation (*n* = 5–11). Statistically significant differences were calculated using Student’s *t* test (* *p* < 0.05; ** *p* < 0.01).

The retraction and swelling capacity of the fibrin scaffolds was analyzed (Figure 4).

In both groups, the retraction ratio percentage increased over time (Figure 4A). At 1 h, sPRP presented a greater retraction capacity, while at 24 h, it exhibited a statistically significantly higher retraction ratio than BPCP (* *p* = 0.041985), indicating a greater contraction of the clot under this condition.

Meanwhile, the swelling ratio was consistently lower in the sPRP group than in the BPCP formulation at multiple time points (Figure 4B). Significant differences were observed at each timepoint (* *p* < 0.05; ** *p* < 0.01), suggesting that the BPCP group retained more fluid and, thus, a higher scaffold weight than sPRP over time (Figure S1, Supplementary Materials).

These results suggest that sPRP exhibits a stronger clot retraction capacity while BPCP demonstrates a greater swelling response over time.

#### 2.3.4. sPRP and BPCP Show a Similar Biodegradation Pattern

**Figure 5:**
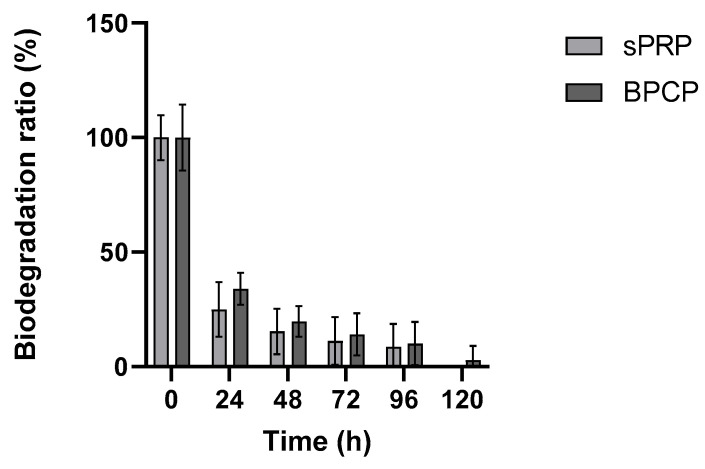
Biodegradation rates of sPRP and BPCP scaffolds. The PRP formulations were exposed to 0.25 μg mL^−1^ tissue plasminogen-activator (tPA) for one week. Error bars = standard deviation (*n* = 5). Statistically significant differences were calculated using Student’s *t* test.

The biodegradation of sPRP and BPCP scaffolds over time following exposure to 0.25 µg mL ^−1^ tissue plasminogen activator (tPA) was measured for five days (Figure 5).

Both groups exhibited a rapid initial degradation, with a steep decline in the biodegradation ratio within the first 24 h. After this period, the degradation rate slowed down considerably, reaching minimal values between 72 and 120 h. No substantial differences were observed between the sPRP and BPCP groups during the experiment, as both formulations followed a similar degradation trend.

These findings suggest that both PRP formulations exhibit a rapid enzymatic degradation phase followed by a stabilization period, indicating similar susceptibility to tPA-mediated breakdown.

### 2.4. Release Kinetics

#### 2.4.1. There Is a Higher Release of Growth Factors by BPCP, Especially on the First Day

Both sPRP and BPCP scaffolds were biochemically characterized by determining their capacity to release GFs over time. Three platelet GFs (TGF-1β, PDGF-AB, and VEGF) and two extraplatelet GFs (IGF-1 and HGF) were analyzed. Overall, BPCP showed a higher GF release compared to sPRP in all patients (Table 3). At 24 h (T_1_), the highest significant differences were found for IGF-1 and the lowest for HGF. In fact, all GFs were significantly higher in BPCP at that timepoint. Meanwhile, TGF-1β levels were significantly higher in BPCP in all patients at all timepoints. Regarding PDGF-AB and VEGF, significant differences were found in most, but not all, cases.

#### 2.4.2. The Total Amount of Released Protein Is Higher in BPCP

**Figure 6:**
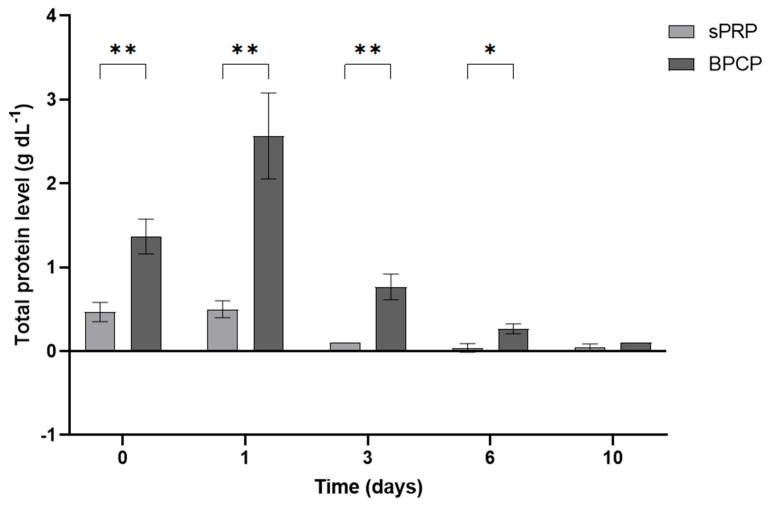
Total protein released from sPRP and BPCP scaffolds. Total protein concentration (g dL^−1^) released from both plasma fibrin clot formulations over a 10-day period are shown. Error bars = standard deviation (*n* = 3). Statistically significant differences were calculated using Student’s *t* test (* *p* < 0.05; ** *p* < 0.01).

In addition to GF levels, the total protein content was analyzed in both scaffolds (Figure 6). BPCP exhibited higher total protein release at each timepoint compared to sPRP. Following 30 min of clot formation (t0), the released protein concentration increased 2.91-fold in BPCP (** *p* = 0.0028). This increase was even more pronounced at 24 h, with a fold change of 5.14 (** *p* = 0.0024). At days 3 and 6, statistically significant differences were still observed (** *p* = 0.0016 and * *p* = 0.0073, respectively). Furthermore, the sPRP scaffold demonstrated a sustained release during the first 24 h (*p* = 0.9799), whereas statistical differences were observed for BPCP (** *p* = 0.0015) (Figure S2, Supplementary Materials).

## 3. Discussion

The regenerative capacity of PRP, once the PRP fibrin clot is completely formed, is based on the release of GFs, which promote processes such as cell proliferation, angiogenesis, migration and reduction of inflammation. However, beyond this biochemical signaling, the fibrin matrix formed during PRP coagulation also plays a crucial biomechanical role by acting as a scaffold for cellular adhesion and migration [28]. PRP is often infiltrated after activation, which leads to platelet aggregation and the reorganization of plasmatic fibrinogen into a well-structured fibrin network across the damaged tissue. As result, a biocompatible fibrin-based scaffold is formed, which enables sustained GF release over time. This matrix not only sustains a localized release of GFs over time but also facilitates cellular anchorage, supporting the structural integrity necessary for tissue repair [29], allowing cell migration, differentiation, proliferation, and adhesion [30].

Altering the composition of PRP could affect its therapeutic effectiveness in the field of regenerative medicine. Several studies have demonstrated that extraplatelet molecules have an impact on the regenerative capacity of PRP [4,23,26,31]. Sanchéz M. et al. [26] have developed a new formulation known as BPCP that demonstrated promising in vitro results. It is characterized by a two-fold increase in both platelet and extraplatelet contents compared to blood levels. Nevertheless, the biochemical and biomechanical properties of the BPCP-based fibrin scaffold have not been studied to date. These characteristics are very important, since when injected directly into living tissues, they undergo shear stress and stretching forces [17].

The present study aimed to analyze the influence of two distinct PRP formulations (sPRP and BPCP) on fibrin scaffold properties, focusing on their biochemical, biomechanical, and biodegradation characteristics, which are crucial to ensure complete tissue regeneration. Our findings revealed key differences between these formulations that have significant implications for their potential applications in regenerative medicine.

The BPCP formulation demonstrated substantially higher fibrinogen content than sPRP, which directly impacted its scaffold properties. The fibrinogen levels in BPCP were approximately double those in sPRP, consistent with previous reports indicating that the fibrinogen concentration plays a crucial role in clot stability and mechanical properties [32,33]. Additionally, both formulations exhibited similar platelet concentrations, ensuring comparable platelet-derived GF levels.

Regarding PRP coagulation kinetics, a controlled fibrin clot formation is crucial for certain applications [34]. In a clotting kinetics analysis, BPCP exhibited a longer clotting time than sPRP. The increase in fibrinolytic proteins such as plasminogen and tPA could increase clotting time since they are responsible of fibrin cleavage [35,36]. This delay may be beneficial for controlled gelation during certain applications, allowing for improved handling during clinical administration. Although it has been shown that a higher concentration of fibrinogen accelerates the coagulation process [33,37], several factors may affect the speed of the process. Among others, the increase of anti-coagulant proteins could play a role in the increased coagulation time. Plasma circulating proteins, like Protein S, Protein C and Thrombomodulin, are responsible for the regulation of the coagulation cascade, inhibiting coagulation factors such as FVIa and VIIIa and thus increasing prothrombin time (PT) [38,39,40,41,42].

Once the fibrin clot was formed after the addition of CaCl_2_ to both PRPs, SEM analysis revealed substantial differences in the fibrin network morphology between BPCP and sPRP scaffold. It has been reported that the fibrinogen and thrombin concentrations influence the thickness and density of fibrin fibers, which subsequently determines the pore size [43]. These characteristics are related to the regenerative effectiveness of the matrices, as they influence other characteristics, which will be discussed later. In the sPRP formulation, the fibrin fibers appeared thinner and more dispersed, with a higher number of visible pores throughout the structure. This porosity suggests a less compact network, which may influence the mechanical properties and stability of the scaffold [33]. In contrast, the BPCP formulation displayed thicker fibrin fibers, resulting in a more robust and denser fibrin network. These results are consistent with the study carried out by Risman RA et al. [44], in which they analyzed the effect of fibrinogen concentration on the structure of clots made from purified fibrinogen and plasma. They found there was an increase in fiber diameter along with a decrease in pore size as the fibrinogen concentration increased in the two formulations. The increased fiber thickness in the BPCP formulation likely contributes to enhanced structural integrity, potentially offering improved mechanical strength and stability compared to the sPRP scaffold.

In terms of clot structure, biomechanical testing further supported these structural differences. BPCP was much more consistent than sPRP, as it exhibited a significantly lower Young’s modulus, indicating lower stiffness and, thus, greater flexibility, as well as a more pronounced energy dissipation capacity compared to sPRP, leading to better cushioning properties [45]. These findings align with research showing that an increased fibrin concentration enhances mechanical integrity [16,22]. Despite these differences, the adhesion strength remained comparable between the formulations, suggesting that both scaffolds maintain sufficient biomechanical stability for tissue engineering applications [37].

The microstructure of the scaffold influences the exchange of biomolecules with the surrounding tissue environment [22]. An important property of fibrin-based scaffolds is their retraction capacity, which is a physiological process within tissue repair. This phenomenon is determined by platelet-generated forces that are transmitted through the fibrin network, which occurs after clot formation, resulting in the reduction of the volume of the clot [46,47]. Platelets interact with the scaffold’s fibrin fibers via the α2bβ3 receptor. After binding, a complex signaling cascade is triggered, which leads to clot retraction [48,49]. In sPRP, the high platelet levels caused platelet aggregation and retraction that prevented the final formation of a stable and homogenous fibrin matrix. Nevertheless, in BPCP, as there is a high level of fibrinogen as well as platelets, less retraction is achieved, resulting in a volume that is more stable over time.

Moreover, pore size is linked to the retraction capacity of scaffolds. The analyses conducted found that BPCP retained significantly more fluid over time, whereas sPRP exhibited a greater tendency toward clot retraction. Coagulation occurs in the plasma fluid itself, and the fluid is retained in the structure. As the pore size is reduced, fluid retention in the matrix is greater. These results are in agreement with the study by Montero A. et al. [50], in which they concluded that increased fibrinogen in plasma-derived fibrin matrices reduced their contractile capacity while improving their mechanical properties. In line with this, Chiu C.L. et al. [43] found that low concentrations of fibrinogen in constructs support greater fluid diffusion. Thus, the lower retraction observed in BPCP suggests a more compact clot structure, which may influence scaffold integration within host tissues.

In the present study, the release of GFs has been analyzed, as the regenerative capacity of PRP relies on the action of these biomolecules. The BPCP scaffold displayed a significantly higher concentration of total proteins, since both the extraplatelet and platelet contents are concentrated. The increase in proteins could be related to a greater release of GFs, especially observed in the first 24 h of the study, in addition to their continuous release over the following 10 days. Moreover, as the days go by, the release of GFs remained higher for BPCP than sPRP, including platelet-derived factors. According to previous studies, GFs released from plasma fibrin scaffolds is sustained for a longer period of time than from other PRPs [51,52,53]. Another reason for the controlled release could be related to the increase in molecular interactions with the heparin sulfate binding domains of fibrin [54,55,56,57], which suggests that BPCP scaffolds could provide more immediate and robust stimulation of tissue regeneration compared to sPRP over time. All this enhances regenerative processes such as cell proliferation, angiogenesis, migration and anti-inflammation [58,59,60,61,62,63].

Regarding how long fibrin matrix degradation takes, our study showed that both PRP formulations exhibited a similar biodegradation pattern upon exposure to tPA. The initial degradation phase was rapid as tPA presents a short half-life [64], followed by a stabilization period, ending with the total degradation of the scaffolds. This suggests that, despite their compositional differences, BPCP and sPRP scaffolds offer comparable degradation rates, which is crucial for balancing scaffold persistence with tissue remodeling dynamics, indicating that the pore size of each scaffold is quite closely related to the GFs and other biomolecules released in a controlled manner.

The present study has several limitations that should be taken into account for future analyses. First, several additional proteins involved in scaffold clotting should be analyzed, such as Protein C, Protein S and coagulation-factors. Second, the in vitro effect of BPCP scaffolds has not been tested. It would be helpful to analyze the effect of the scaffold on cell proliferation and adhesion, as well as cell encapsulation and anti-inflammatory capacity by measuring the pro- and anti-inflammatory profile of each scaffold. Furthermore, it would be useful to measure the release of other GFs and/or plasma proteins involved in regenerative processes and explore how these proteins affect the fibrin architecture and cell behavior. Another limitation of this work is the amount of blood required for each assay that limits the possibility of increasing the sample size, thus increasing the variability of the assays. Finally, in vivo and clinical trials are required to evaluate the behavior of BPCP scaffolds inside the organism and assess factors like new tissue development, scar formation, safety, and efficacy.

These findings will open the door for innovative approaches and further research on the development of PRP-derived autologous scaffolds. Future studies could concentrate on translational and clinical research, exploring the use of this matrix in treating various pathological conditions, such as wounds or osteochondral defects.

## 4. Materials and Methods

### 4.1. Sample Collection and Preparation

#### 4.1.1. Donors

Healthy volunteers aged between 18 and 65 years were selected for this study. Blood was collected in 9 mL and 3.5 mL tubes, both containing 3.8% (*w*/*v*) sodium citrate. The 3.5 mL tubes were used for baseline platelet concentration measurements, while the 9 mL tubes were used to prepare several plasma formulations. This research was carried out following the principles outlined in the Declaration of Helsinki and was approved by the Institutional Ethics Committee of OSI Araba (approval number 2024-023, dated 30 May 2024). Written informed consent was obtained from all participants prior to their involvement in the study.

#### 4.1.2. Standard Platelet-Rich Plasma Preparation

Briefly, the sPRP was obtained by using a commercially available PRP kit (BTI Biotechnology Institute, Vitoria-Gasteiz, Spain) by centrifuging 9 mL of blood at 580× *g* for 8 min at room temperature (RT). Then, 2 mL of the plasma fraction present over the red blood cell fraction was collected, avoiding white blood cells from the ‘buffy coat’ layer.

Finally, 10% CaCl_2_ (20 μL mL^−1^) was added to the sPRP formulation and it was kept at 37 °C for the release of platelet content and scaffold formation.

#### 4.1.3. Balanced Protein-Concentrate Plasma Preparation

As described by Mercader et al. [31], the BPCP was obtained by centrifuging 9 mL of whole blood at 1200× *g* for 8 min at RT, collecting the entire plasma column (PC). The obtained plasma fraction contained levels of platelets and circulating molecules similar to blood basal levels. It was put in contact with 0.125 g mL^−1^ of HEAA hydrogel. After 5 min, the plasma’s water content was absorbed and the hydrogel powder was discarded by putting the plasma in contact with a 100 µm filtration unit on top of a 50 mL falcon tube. Centrifugation at 500× *g* for 2 min at RT was performed to collect the BPCP.

Finally, 10% CaCl_2_ (20 μL mL^−1^) was added to the BPCP formulation and it was kept at 37 °C for the release of platelet content and scaffold formation.

### 4.2. Platelet and Fibrinogen Level Measurement

Platelet and fibrinogen levels were measured in blood, sPRP and BPCP. For the analysis of the platelet content, a Mindray BC-20s hematology analyzer (Mindray, Shenzhen, China) was used, whereas fibrinogen levels were measured using a coagulation analyzer (STA Compact Max, Stago, Asnières-sur-Seine, France).

### 4.3. Morphological Analysis

Microscopic analysis of the ultrastructure of the sPRP and BPCP scaffolds was carried out using scanning electron microscopy (SEM) (Hitachi S-4800; Hitachi, Tokyo, Japan) by the SGIKER service at the University of the Basque Country (UPV/EHU). Once the fibrin clots were formed, they were rinsed with PBS for 30 min and fixed using 2% glutaraldehyde (1121790025; Sigma–Aldrich, Darmstadt, Germany) in Sorensen’s Buffer (11682-10-4L; Quimigen, Alverca do Ribatejo, Portugal). Treatment with osmium tetroxide (OsmO4) and critical-point-dried steps were carried out by the SGIKER service. Fibrin fiber diameter and pore size was measured with ImageJ/FIJI (National Institute of Health, MD) software following a detailed protocol based on a previous study [65].

### 4.4. Biomechanical Behavior

#### 4.4.1. Coagulation Kinetics

Clotting time for sPRP and BPCP was measured by turbidimetry at 450 nm using the TECAN Infinite 200 PRO plate reader (TECAN, Zurich, Switzerland). Both PRP formulations were activated and, subsequently, 100 µL of each formulation was added to a 96-well plate (3628; Sigma–Aldrich). Absorbance was measured every 2 min for 1 h to ensure clot formation. Technical triplicates were carried out.

#### 4.4.2. Mechanical Tests

To analyze the biomechanical properties of the PRP clots, Young’s modulus, dissipated energy and adhesion capacity were measured. To determine Young’s modulus and the dissipated energy of the sPRP and BPCP, instrumented indentation tests were carried out using a spherical indenter. A calibrated spherical indenter with a diameter of 5 mm was pressed onto the sample, recording the load used to penetrate the indenter as well as the penetration distance at each moment. The sphere penetrated the sample at a constant speed of 50 µm s^−1^ until it reached a depth equivalent to 20% of the initial thickness of the sample. Once this penetration was reached, the direction of movement was reversed, discharging the indenter. The indentations were performed directly on 24-well plates (3524; Sigma–Aldrich), with scaffolds of 2 mm in thickness. Measurements were carried out at three different locations on each clot. An empty well was used as a blank.

Regarding the adhesiveness of both types of matrices, two cylinders were covered with a polypropylene gauze and impregnated with 100 µL of the formulation previously activated. Then, the two gauzes were pressed together (30 mN) and the necessary time was allowed for complete coagulation. After the formation of the matrices, an attempt was made to detach the cylinders by separating the two glued gauzes at a constant speed of 3 mm min^−1^. The resulting adhesion strength was calculated by identifying the area of the glued gauzes and the maximum force required for their separation during the test.

All tests were performed using a Zwick/Roell ZwickiLine Z1.0 uniaxial testing machine (Ulm, Germany) by the Research Management Service of the University of Navarra (UNAV, Donostia-San Sebastian, Spain). The load cell used was a Zwick/Roell Xforce P with a maximum load of 50 N.

#### 4.4.3. Rheological Profile: Amplitude Sweep Oscillatory Test

An MCR 301 rheometer (Anton Paar GmbH, Graz, Austria) was used by the Research Management Service of the University of Navarra (UNAV, Donostia-San Sebastian, Spain) to assess the viscoelastic behavior of the sPRP and BPCP formulations. Matrices of 1 mL of PRP previously activated were formed in 24-well plates to ensure a clot diameter of 8 mm. The test was performed at RT with an oscillatory frequency of 10 rad s ^−1^ and a strain range of 0.1% to 100%, starting with an initial force of 0 N. The sample thickness was set to 0.5 mm, and the sweep included 25 points within the strain range. The results were analyzed based on the linear viscoelasticity (LVE) criterion, where the relative variation in the storage modulus (G′) and loss modulus (G″) was kept below 5% in accordance with ISO standards.

#### 4.4.4. Swelling and Retraction

Swelling and retraction properties were determined using a gravimetric method. In order to prepare sPRP and BPCP scaffolds, 500 µL of both formulations were activated in duplicate in 3.5 mL tubes and incubated at 37 °C for 30 min to allow complete coagulation.

After clot formation, the scaffolds were weighed (t_0_), and then incubated in 1 mL PBS at 37 °C to allow any swelling or shrinkage to occur. After one hour and every 24 h thereafter, the samples were placed in PBS and the clots were weighed. Each time, after weight measurement, the clots were immersed in fresh PBS. This process was performed for 2 weeks. The swelling percentage was calculated by comparing the weight of each time point to the initial weight by the following equation:

(1) $$\mathrm{Swelling}\;\%=\left((W-W_{0}\right)/W_{0})\times 100$$

The retraction percentage was calculated by comparing the initial weight of the clot to its final weight after a specific period of time using the following equation:

(2) $$\mathrm{Retraction}\;\%=\left((W_{0}-W\right)/W_{0})\times 100$$

This formula calculates the percentage of weight loss due to clot retraction, measuring the degree to which the clot has contracted over time.

#### 4.4.5. Biodegradation

Degradation upon exposure to tissue plasminogen activator (tPA) was studied over a period of 5 days by measuring mass loss. tPA activates the conversion of plasminogen into plasmin, which is responsible for the degradation of plasmatic fibrin in vivo. In this assay, tPA is just added to speed up the fibrin clot biodegradation [17]. The sPRP and BPCP scaffolds were weighed (t_0_) 1 h after activation. Subsequently, they were refreshed with PC containing tPA (0.25 µg ml^−1^, Abcam, Cambridge, UK) and they were kept at 37 °C. Weight loss was recorded every 24 h with tPA refreshment but no PC. Assays were performed using plasma samples from five donors and technical triplicates were carried out.

### 4.5. Release Kinetics

sPRP and BPCP were placed into a 12-well plate and incubated at 37 °C for 30 min until coagulation. Once the scaffolds were formed, excess plasma was removed and 3 mL of Dulbecco’s modified Eagle medium (DMEM, Gibco-Invitrogen, Grand Island, NY, USA) was added to each well. The plates were incubated again at 37 °C. The culture medium was collected and replaced at specific time points (days 0, 1, 3, 6, and 10) and stored at −80 °C for further analysis. Assays were performed using samples from three different donors.

#### Platelet and Extraplatelet Growth Factor Concentration Measurements by Enzyme-like Immunosorbent Assay (ELISA) and Total Protein Release Analysis

The concentrations of platelet and extraplatelet molecules in the collected DMEM were measured using ELISA assays. The following proteins were measured in accordance with the manufacturer’s instructions: VEGF (DVE00; Bio-techne, Minneapolis, MI, USA), TGF-β1 (DB100C; Bio-techne), IGF-1 (DG100B; Bio-techne), HGF (DHG00B; Bio-techne) and PDGF-AB (DHD00C; Bio-techne). All protein levels were measured by absorbance and the corresponding concentrations were calculated by means of calibration curves (4PL).

The total protein level released into the DMEM was measured using a Cobas c 501 analyzer (Roche, Basel, Switzerland).

### 4.6. Statistical Analysis

The distribution of the data was assessed by Shapiro–Wilk’s normality test. The different variables are presented as the means and the standard deviations for parametric data. The statistical significance of the differences between groups were determined with Student’s *t*-test. Data were considered statistically significant when *p* < 0.05. GraphPad Prism^®^ software 9.5 version (San Diego, CA, USA) was used for the statistical analyses.

## 5. Conclusions

The differences observed between the sPRP and BPCP scaffolds provide valuable insights for optimizing PRP formulations in regenerative medicine. The enhanced GF release, structural integrity, and prolonged swelling behavior of BPCP suggest its potential superiority in applications requiring sustained tissue regeneration, such as chronic wound healing and orthopedic tissue repair. However, further in vivo studies are necessary to confirm the clinical efficacy of these scaffolds. Future research should focus on tailoring PRP formulations to specific clinical needs by modulating the fibrinogen concentration and clot structure.

## Figures and Tables

**Table 1:** Summary of characteristics for both formulations of PRP.

	sPRP	BPCP
**1.** ** ** **PRP Preparation**		
Initial blood volume	9 mL per tube	9 mL per tube
Anticoagulant	Sodium citrate 3.8% (wt V^−1^)	Sodium citrate 3.8% (wt V^−1^)
System	Closed	Open
Centrifugation	Yes	Yes
Number	1	1
Speed	580× *g*—8 min	1200× *g*—8 min
Water absorption	No	Yes
Method	---	HEAA hydrogel
Hydrogel concentration	---	0.125 g mL^−1^
Contact time	---	5 min
Final PRP volume	2 mL per tube	2 mL per subject
**2.** ** ** **PRP Characteristics**		
PRP Type	13-00-11 [27]	13-00-11 [27]
Platelets	335.76 × 10^3^ µL^−1^ ± 111.32	342.94 × 10^3^ µL^−1^ ± 117
Fibrinogen	364.57 mg dL^−1^ ± 32.56	718.71 mg dL^−1^ ± 55.95
Red Blood Cells	<0.01 × 10^6^ µL^−1^	<0.01 × 10^6^ µL^−1^
White Blood Cells	<0.05 × 10^6^ µL^−1^	<0.05 × 10^6^ µL^−1^
Neutrophils	---	---
Lymphocytes	---	---
Monocytes	---	---
Eosinophils	---	---
Basophils	---	---
Activation	CaCl_2_ (10% wt vol^−1^)	CaCl_2_ (10% wt vol^−1^)
**3.** ** Application Characteristics**		
Dose	10%	10%
Direct/Indirect	Direct	Direct
**4.** ** Other remarkable PRP and study features**		
The product used for the study was the fibrin clot obtained following the activation of PRP with calcium chloride (10%).		

sPRP: Standard platelet-rich plasma; BPCP: Balanced protein-concentrate plasma; HEAA: Hydroxyethyl acrylamide.

**Table 2:** Rheological and biomechanical properties of sPRP and BPCP scaffolds.

		sPRP	BPCP	*p* Value
**Rheological properties**	Elastic modulus (G′) (kPa)	422.41 ± 280.05	588.89 ± 383.9	0.3722
	Viscous modulus (G″) (kPa)	99.31 ± 69.07	131.76 ± 95.32	0.4797
	Tanδ	0.25 ± 0.08	0.21 ± 0.06	0.5350

Rheological properties were assed at RT with an oscillatory frequency of 10 rad s^−1^ and a strain range from 0.1 to 100%, starting with an initial force of 0 N.

**Table 3:** Release kinetics (pg mL^−1^) of both extraplatelet and platelet derived GFs for 10 days.

	P1			P2			P3		
	sPRP	BPCP	*p* Value	sPRP	BPCP	*p* Value	sPRP	BPCP	*p* Value
***T_0_***									
TGF-β1	1357 ± 47.68	2048 ± 75.64	0.008 **	1543 ± 26.61	3302 ± 33.17	0.0003 ***	3724 ± 20.71	4060 ± 8.98	0.002 **
PDGF-AB	371 ± 3.99	592 ± 12.25	0.002 **	438 ± 2.24	964 ± 28.66	0.0015 **	1139 ± 52.52	1132 ± 27.61	0.88
VEGF	6.5 ± 0.21	15 ± 1.57	0.016 *	16 ± 0.87	38 ± 1.78	0.004 **	18 ± 1.30	21 ± 0.00	0.11
IGF-1	5 ± 0.10	16 ± 0.41	0.0007 ***	6 ± 0.10	28 ± 0.01	<0.0001 ****	8 ± 0.08	17 ± 0.05	<0.0001 ****
HGF	12.5 ± 0.00	52 ± 6.24	0.12	10 ± 1.89	55 ± 0.12	0.0009 ***	32 ± 3.06	86.5 ± 4.07	0.004 **
***T*_1_ (24 h)**									
TGF-β1	2480 ± 68.84	3902 ± 39.76	0.0015 **	2877 ± 52.15	5645 ± 166.10	0.002 **	4057 ± 33.96	4973 ± 55.49	0.003 **
PDGF-AB	620 ± 22.04	1189 ± 2.80	0.0008 ***	669 ± 0.73	1874 ± 24.59	0.0002 ***	1347 ± 6.89	2313 ± 71.58	0.003 **
VEGF	17 ± 0.33	37 ± 0.29	0.0002 ***	37 ± 0.24	81 ± 3.19	0.003 **	50 ± 0.43	60 ± 1.00	0.006 **
IGF-1	7 ± 0.14	26 ± 0.02	<0.0001 ****	6.5 ± 0.24	45 ± 1.16	0.0005 ***	7 ± 0.46	23 ± 0.15	0.0004 ***
HGF	21 ± 2.48	105 ± 11.98	0.0104 *	7 ± 0.48	101 ± 15.17	0.013 *	39 ± 3.33	252 ± 14.94	0.003 **
***T*_3_ (72 h)**									
TGF-β1	593 ± 23.13	2157 ± 87.37	0.002 **	773 ± 30.58	1890 ± 16.69	0.0005 ***	1761 ± 0.36	3306 ± 137.54	0.004 **
PDGF-AB	149 ± 0.15	515 ± 46.15	0.009 **	189 ± 12.83	516 ± 9.56	0.0012 **	439 ± 6.76	872 ± 13.92	0.0006 ***
VEGF	0.5 ± 0.32	14 ± 0.16	0.0003 ***	10 ± 0.19	19 ± 0.24	0.0005 ***	19 ± 0.52	15 ± 1.48	0.051
IGF-1	0.8 ± 0.00	10.5 ± 0.42	0.0009 ***	1 ± 0.04	10 ± 0.00	<0.0001 ****	1 ± 0.01	9 ± 0.11	0.0001 ***
HGF	9 ± 12.27	34 ± 0.80	0.102 *	4 ± 0.00	15 ± 4.45	0.31	6 ± 8.30	48 ± 2.34	0.02 *
***T*_6_ (144 h)**									
TGF-β1	333 ± 8.33	902 ± 40.19	0.003 **	705 ± 15.10	1232 ± 37.91	0.003 **	943 ± 9.84	2748 ± 123.57	0.002 **
PDGF-AB	66 ± 1.47	208 ± 1.59	0.00012 ***	119 ± 1.60	256 ± 10.13	0.003 **	247 ± 5.03	639 ± 7.34	0.0002 ***
VEGF	0 ± 0.00	1 ± 0.40	0.037 *	3 ± 0.16	6 ± 0.00	0.001 **	8 ± 4.38	7 ± 0.38	0.78
IGF-1	0.5 ± 0.00	2 ± 0.04	0.0004 ***	0.5 ± 0.00	3 ± 0.02	<0.0001 ****	0.6 ± 0.03	3 ± 0.02	0.00012 ***
HGF	0.2± 0.12	8.5 ± 2.04	*0.029*	2 ± 1.01	5 ± 0.73	0.12	0 ± 0.00	18.5 ± 6.34	*0.053*
***T*_10_ (240 h)**									
TGF-β1	586 ± 5.70	820 ± 10.72	0.0013 **	462 ± 23.21	1654 ± 26.53	0.0004 ***	724 ± 1.49	1900 ± 16.31	<0.0001 ****
PDGF-AB	48 ± 1.35	103 ± 4.04	0.003 **	82 ± 4.61	163 ± 2.88	0.002 **	106 ± 0.63	345 ± 3.22	<0.0001 ****
VEGF	0.3 ± 0.31	0 ± 0.00	0.26	0 ± 0.00	4 ± 0.30	0.003 **	1 ± 0.62	4 ± 0.81	0.09
IGF-1	0.5 ± 0.00	0.5 ± 0.01	0.063	0.5 ± 0.01	0.3 ± 0.02	0.005 **	0.6 ± 0.02	0.7 ± 0.04	0.24
HGF	2 ± 0.97	4 ± 0.00	0.29	1 ± 0.37	0 ± 0.00	0.11	7 ± 5.00	10 ± 0.00	*0.42*

GF: Growth factor; P: Patient; T: Time (days); sPRP: Standard platelet-rich plasma; BPCP: Balanced protein-concentrate plasma; TGF-β1: Transforming growth factor beta-1; PDGF-AB: Platelet-derived growth factor-AB; VEGF: Vascular endothelial growth factor; IGF-1: Insulin-like growth factor-1; HGF: Hepatocyte growth factor. Mean and standard deviation values are represented in each timepoint. Statistically significant differences were calculated using Student’s *t* test (*n* = 3). Statistically significant data is represented in bold and italics; * *p* < 0.05; ** *p* < 0.01; *** *p* < 0.001; **** *p* < 0.0001.

## Data Availability

Data will be made available on request.

## Supplementary Materials

The following supporting information can be downloaded at: https://www.mdpi.com/article/10.3390/ijms26135967/s1.

## Abbreviations

The following abbreviations are used in this manuscript:

A2M	Alpha-2-macroglobulin
BPCP	Balanced protein concentrate plasma
GF	Growth factor
HEAA	Hydroxyethyl acrylamide
HGF	Hepatocyte growth factor
IGF-1	Insulin-like growth factor 1
LVE	Linear viscoelasticity
OsO4	Osmium tetroxide
PC	Plasma column
PDGF-AB	Platelet-derived growth factor AB
PRP	Platelet-rich plasma
RT	Room temperature
SEM	Scanning electron microscope
sPRP	Standard platelet-rich plasma
TGF-β1	Transforming growth factor beta 1
tPA	Tissue plasminogen activator
VEGF	Vascular endothelial growth factor
