# Supplementary material for: Exploring the Impact of Extraplatelet Content on Fibrin-Based Scaffold Performance for Regenerative Therapies
Source: Int J Mol Sci. 2025 Jun 21;26(13):5967. doi: 10.3390/ijms26135967 (PMC12249790; doi:10.3390/ijms26135967)
Supplement: Supplementary file 1 [file ijms-26-05967-s001.zip › ijms-3680775-supplementary.pdf]

Supplementary Material

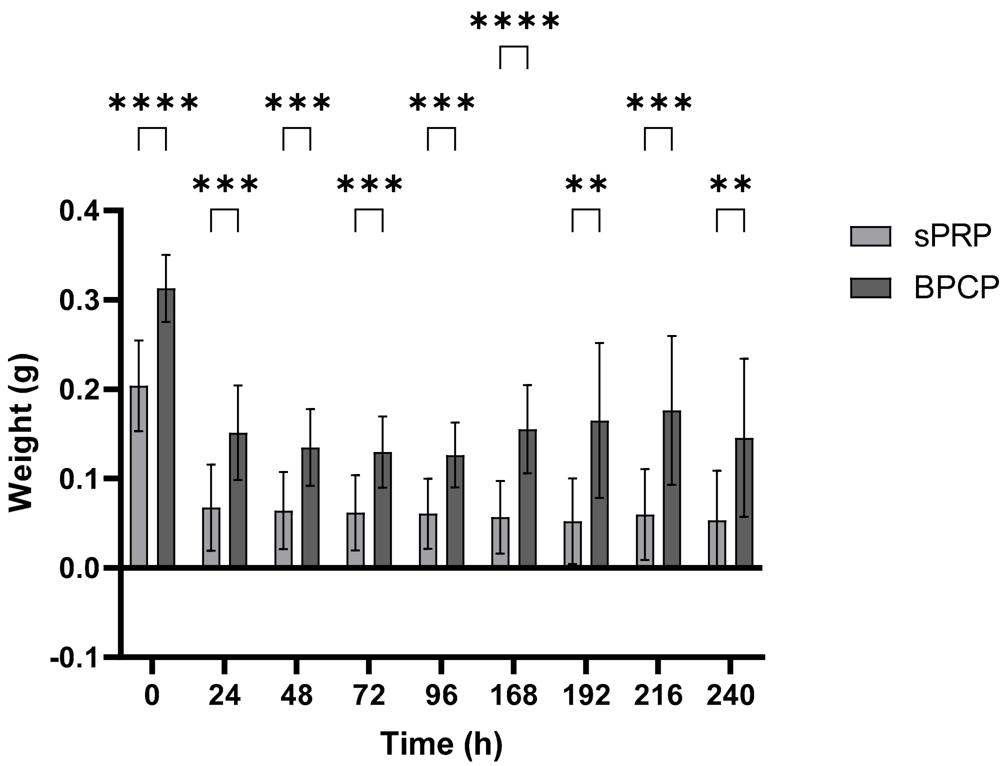

Figure S1: sPRP and BPCP scaffold's weight for two weeks. Scaffolds of 500 µL of sPRP or BPCP were made and incubated in 1 mL of PBS at 37ºC for two weeks. Every 24 h clots were weighted and PBS was removed. Error bars = standard deviation (*n* = 11). Statistically significant differences were calculated using Student's t-test (** $p < 0.01$; *** $p < 0.001$; **** $p < 0.0001$).

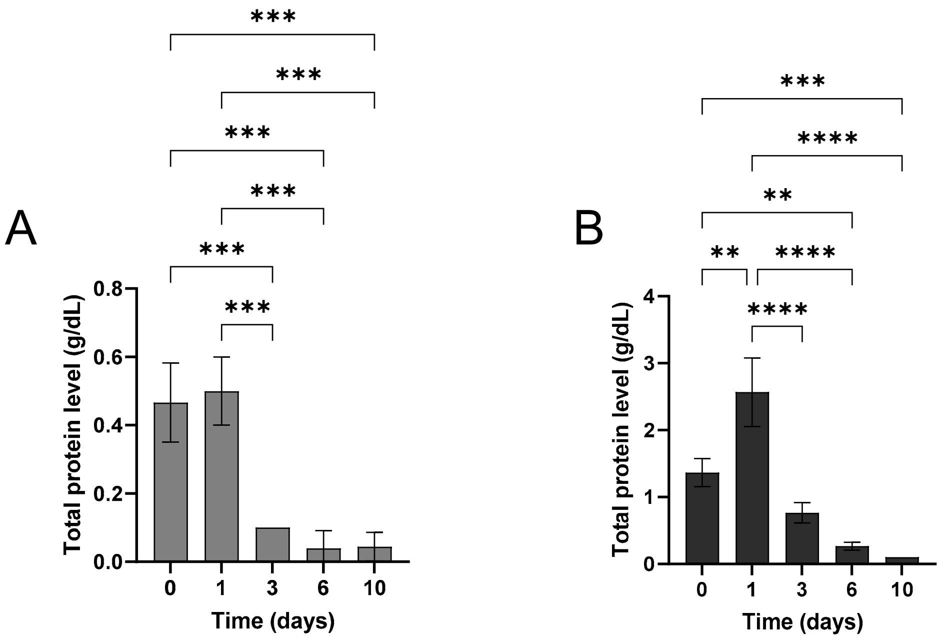

Figure S2: Released total protein levels over time. Total protein concentration released from sPRP scaffold (A) and BPCP scaffold (B) for 10 days. Error bars = standard deviation (*n* = 3). Statistically significant differences were calculated using Ordinary ANOVA one-way analysis (** $p < 0.01$; *** $p < 0.001$; **** $p < 0.0001$).
